# Molecular Detection and Genetic Identification of *Rickettsia* Infection in *Ixodes granulatus* Ticks, an Incriminated Vector for Geographical Transmission in Taiwan

**DOI:** 10.3390/microorganisms9061309

**Published:** 2021-06-16

**Authors:** Chien-Ming Shih, Pei-Wen Yang, Li-Lian Chao

**Affiliations:** 1 M.Sc. Program in Tropical Medicine, College of Medicine, Kaohsiung Medical University, Kaohsiung 807, Taiwan; cmshih@kmu.edu.tw; 2 National Defense Medical Center, Graduate Institute of Pathology and Parasitology, Taipei 114, Taiwan; peiwei54@hotmail.com; 3 Department of Medical Research, Kaohsiung Medical University Hospital, Kaohsiung 807, Taiwan

**Keywords:** *Rickettsia*, *Ixodes granulatus*, tick, genetic identity, Taiwan

## Abstract

Tick-borne *Rickettsia* pathogens have become an emerging source of zoonotic infections and have a major impact on human health worldwide. In this study, the prevalence and genetic identity of *Rickettsia* infections in *Ixodes granulatus* ticks was firstly determined in Kinmen Island of Taiwan. A total of 247 *I. granulatus* ticks were examined for *Rickettsia* infection by nested-PCR assay targeting the citrate synthase (gltA) gene of *Rickettsia*. The *Rickettsia* infection was detected with a general infection rate of 4.86%, and was detected in nymph, male and female stages with an infection rate of 3.81%, 0% and 6.84%, respectively. Phylogenetic relationships were analyzed by comparing the gltA sequences obtained from four Taiwan strains and 19 other strains representing 13 genospecies of *Rickettsia*. Phylogenetic analyses reveal that all Taiwan strains were genetically affiliated to the genospecies of spotted fever (*R. parkeri*) and transitional (*R. felis*) groups of *Rickettsia*. Our findings reveal the first detection of *R. parkeri*-like and *R. felis* in *I. granulatus* ticks from Kinmen Island. As a tourist island between Taiwan and mainland China, these results demonstrate the epidemiological significance of diverse *Rickettsia* species existed in *I. granulatus* ticks and highlight the potential threat of geographical transmission among humans in the Taiwan area.

## 1. Introduction

The genus *Rickettsia* is composed of approximately 27 species of obligate intracellular gram-negative bacteria that can be classified into four major groups: ancestral group (AG), typhus group (TG), transitional group (TRG) and spotted fever group (SFG) [1,2,3]. The Ixodid ticks may serve as vectors and reservoirs of amplifying hosts for *Rickettsia* agents [4]. The SFG rickettsiae are mainly transmitted by vector ticks that can transmit the *Rickettsia* agents through the transovarial and transstadial pathways [5,6]. During recent decades, *Rickettsia* infections have become a global threat for emerging and re-emerging tick-borne diseases [7,8]. Global climate change has enhanced the geographical distribution of ticks and also expanded the spread of tick-borne pathogens [9,10]. Most SFG rickettsioses are found in a particular geographic location [10,11,12,13,14,15,16], and many validated SFG rickettsial species have been discovered in Australia, Central and South America, and Asia [17,18,19,20,21,22,23,24,25]. Thus, an epidemiological survey on tick-borne rickettsiae is important to understand the potential threat of emerging and re-emerging tick-borne *Rickettsia* infections in the Taiwan area.

Ticks are obligate hematophagous arthropod that may act as vectors with the ability to transmit various pathogens including bacteria, rickettsiae, protozoans and viruses [26]. The abundance and spread of *Ixodes granulatus* ticks had been recorded from various countries in Southeast Asia and Taiwan [27]. This tick species is found mainly on rodent hosts captured in grassy areas and vegetable gardens [28]. The medical and veterinary importance of the molecular detection of Lyme disease spirochetes (*Borrelia burgdorferi* sensu lato) and related spirochete (*Borrelia valaisiana*) has been identified in various stages of *I. granulatus* ticks of Taiwan [28,29]. In addition, the *Rickettsia* species of spotted fever group rickettsia had also been identified in *I. granulatus* ticks collected from Japan and offshore Orchid Island (Lanyu) of Taiwan [30,31]. Although the *I. granulatus* ticks have been recognized as the incriminated vector ticks for a variety of pathogens in Taiwan, there has no research demonstrating the prevalence of *Rickettsia* infection and confirming the genetic diversity in this tick species in Taiwan.

A DNA-based approach provides the feasibility to investigate the genetic variance at the individual base-pair level and gives a much more direct pathway for measuring the genetic diversity between and within species of *Rickettsia* [32]. Previous studies based on the molecular marker of citrate synthase gene (*gltA*) have concluded that it is sufficiently informative for the analysis of evolutionary relationships between the genetic diversity of *Rickettsia* species among various vectors and hosts [16,22,23,33]. Thus, molecular detection and genetic analysis based on the phylogenetic analysis of *gltA* gene has facilitated the identification and discrimination of *Rickettsia* species within ticks.

It may be that the *Rickettsia* infection in *I. granulatus* ticks of Taiwan is a genetically distinct genospecies, as compared with the existing common genospeciess of *Rickettsia* around the world. Thus, the objectives of this study intend to investigate the prevalence of *Rickettsia* infection in *I. granulatus* ticks collected from offshore Kinmen Island of Taiwan, and to determine the phylogenetic relationships between and within the genospecies of *Rickettsia* in these ticks. The genetic affiliation of *Rickettsia* strains detected in *I. granulatus* ticks of Taiwan was analyzed by comparing their differential nucleotide composition with other *Rickettsia* strains identified from various biological and geographical sources which have been documented in GenBank.

## 2. Materials and Methods

### 2.1. Tick Collection and Species Identification

**Figure 1:**
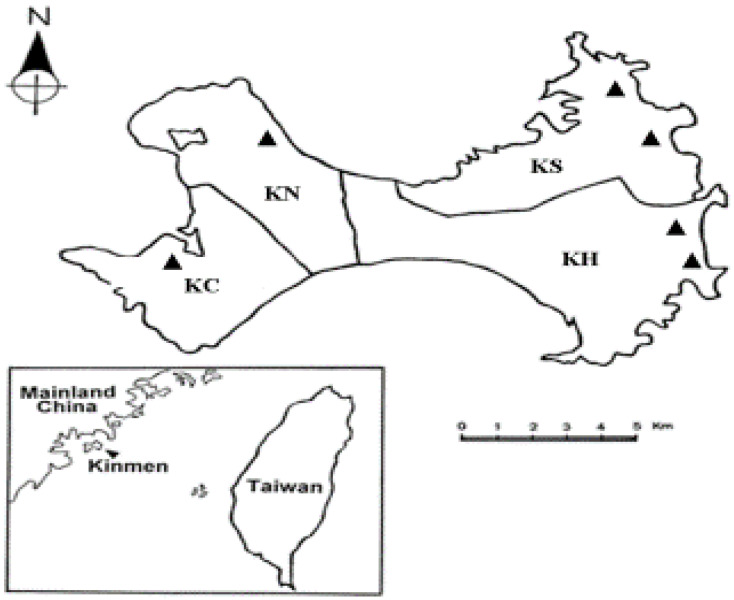
Map of Kinmen Island showing the various collection sites (indicated as ▲) for ticks removed from trapped rodents.

All specimens of *I. granulatus* ticks used in this study were collected from rodents captured in the grassy areas and vegetable gardens, especially the peanut and sweet potato. The collection sites in four townships of Kinmen Island include Kinhu (24°41′ N, 118°43′ E; 24°43′ N, 118°46′ E), Kinsha (24°52′ N, 118°41′ E; 24°50′ N, 118°44′ E), Kinning (24°45′ N, 118°37′ E) and Kincheng (24°40′ N, 118°31′ E) (Figure 1).

All these ticks were subsequently cleaned and stored in separate glass vials containing 75% ethanol. All tick specimens of *I. granulatus* were identified to the species level on the basis of their morphological characteristics, as described previously [28]. Briefly, the external features of the *I. granulatus* ticks were recorded by using a stereo-microscope (SMZ 1500, Nikon, Tokyo, Japan) equipped with a fiber lamp and photographed for species identification.

### 2.2. DNA Extraction from Tick Specimens

Total genomic DNA was extracted from individual tick specimens used in this study. Briefly, tick specimens were cleaned by sonication for 3–5 min in 75% ethanol solution and then washed twice in sterile distilled water. Afterwards, the individual tick specimen was homogenized in a microcentrifuge tube filled with 180 μL lysing buffer solution (DNeasy Blood &Tissue Kit, catalogue no. 69506, Qiagen, Taipei, Taiwan) and then homogenized with a TissueLyser II apparatus (catalogue no. 85300, Qiagen, Taipei, Taiwan Branch), instructed by the manufacturer. The homogenate was centrifuged at room temperature and the supernatant fluid was further processed by a DNeasy Tissue Kit, as instructed by the manufacturer. After filtration, the filtrated fluid was collected and the DNA concentration was determined spectrophotometrically with a DNA calculator (Epoch, Biotek, Taipei, Taiwan Branch) and the extracted DNA was stored at −80 °C for further investigations.

### 2.3. DNA Amplification by Nested Polymerase Chain Reaction

DNA samples extracted from each tick specimens were used as a template for PCR amplification. Two primer sets based on the citrate synthase gene (*gltA*) were used for amplification. Initially, the primer set of RpCS.877p (5′-GGGGGCCTGCTCACGGCGG-3′) and RpCs.1258n (5′-ATTGCAAAAAGTACAGTGAACA-3′) was used to amplify the primary product of *gltA*. Nested PCR was then performed using the species-specific primer sets: RpCS.896p (5′-GGCTAATGAAGCAGTGATAA-3′) and RpCS.1233n (5′-GCGACGGTATACCCATAGC-3′) for amplifying a product approximately 338 bp [11]. All PCR reagents and Taq polymerase were obtained and used as recommended by the supplier (Takara Shuzo Co., Ltd., Kyoto, Japan). Briefly, each 25 μL reaction mixture contained 3 μL DNA template, 1.5 μL forward and reverse primers, 2.5 μL 10X PCR buffer (Mg^2+^), 2 μL dNTP mixture (10 mM each), 1 unit of Taq DNA polymerase and was filled-up with adequate volume of ddH_2_O. In contrast, adequate amounts of sterile distilled water were added for serving as a negative control. PCR amplification was performed with a thermocycler (Veriti, Applied Bioosystems, Taipei, Taiwan) and was denatured at 95 °C for 5 min and then amplified for 35 cycles with the conditions of denaturation at 95 °C for 30 s, annealing at 54 °C for 30 s, extension at 72 °C for 1 min, and followed by a final extension step at 72 °C for 3 min. For the nested-PCR, the following conditions were used: denaturation at 95 °C for 5 min and then amplified for 40 cycles with the conditions of denaturation at 95 °C for 30 s, annealing at 50 °C for 30 s, extension at 72 °C for 1 min, and followed by a final extension step at 72 °C for 3 min.

For outer membrane protein B gene (*ompB*), same quantities as in *gltA* for the reaction mixture were used. The primer sets of rompB-OF (5′-GTAACCGGAAGTAATCGTTCGTAA-3′) and rompB-OR (5′-GCTTTATAACCAGCTAAACCACC-3′) was used to amplify the primary product of *ompB*. Nested PCR was then performed using the species-specific primer sets: rompB SPG-IF (5′-GTTTAATACGTGCTGCTAACCAA-3′) and rompB SPG/TG-IR (5′-GGTTTGGCCCATATACCATAAG-3′) for amplifying a product approximately 420-bp [11]. The PCR conditions were used same as *gltA* amplification except the annealing temperature of 50 °C and 52 °C for the initial and nested PCR cycles, respectively.

All amplified PCR products were electrophoresed on 1.5% agarose gels in Tris-Borate-EDTA (TBE) buffer and visualized under ultraviolet (UV) light after staining with ethidium bromide. A 100-bp DNA ladder (GeneRuler, Thermo Scientific, Taipei, Taiwan) was used as the standard marker for comparison. A negative control of distilled water was included in parallel with each amplification.

### 2.4. Sequence Alignments and Phylogenetic Analysis

Approximately 10-μL of each selected samples with clear bands on the agarose gel was submitted for DNA sequencing (Mission Biotech Co., Ltd., Taipei, Taiwan). After purification (QIAquick PCR Purification Kit, catalog No. 28104), sequencing reaction was performed with 25 cycles under the same conditions and same primer set of nested amplification by dye-deoxy terminator reaction method using the Big Dye Terminator Cycle Sequencing Kit in an ABI Prism 377-96 DNA Sequencer (Applied Biosystems, Foster City, CA, USA). The resulting sequences were initially edited by BioEdit software (V5.3) and aligned with the CLUSTAL W software [34]. Thereafter, the aligned sequences of *Rickettsia gltA* gene from four Taiwan strains were analyzed by comparing with other 19 strains of *Rickettsia* sequences from the different biological and geographical origin that are available from GenBank. Further analysis based on *ompB* genes of two Taiwan strains belonging to the SFG *Rickettsia* was performed by comparing with other 14 strains of *Rickettsia* sequences documented in GenBank. Phylogenetic analysis was performed by neighbor-joining (NJ) compared with maximum likelihood (ML) methods to estimate the phylogeny of the entire alignment using MEGA X software package [35]. The genetic distance values of inter- and intra-species variations were also analyzed by the Kimura two-parameter model [36]. All phylogenetic trees were constructed and performed with 1000 bootstrap replications to evaluate the reliability of the construction, as described previously [37].

### 2.5. Nucleotide Sequence Accession Numbers

The nucleotide sequences of PCR-amplified *gltA* genes of four *Rickettsia* strains from *I. granulatus* ticks of Taiwan determined in this study have been registered and assigned the following GenBank accession numbers: Ig-9103-KH-56 (MT847612), Ig-9103-KH-47 (MT847613), Ig-9106-KH-100 (MT847614) and Ig-9012-KS-495-N (MT847619). The GenBank accession numbers for the PCR-amplified *ompB* genes of two *Rickettsia* strains from *I. granulatus* ticks of Taiwan were also assigned as: Ig-9103-KH-47 (MZ198103) and Ig-9106-KH-100 (MZ198104). For phylogenetic analysis, the nucleotide sequences of *gltA* and *ompB* genes from other 19 and 14 *Rickettsia* strains were included for comparison, respectively. Their GenBank accession numbers are shown in Table 1.

## 3. Results

### 3.1. Detection of Rickettsia in I. granulatus Ticks from Taiwan

The existence of *Rickettsia* in *I. granulatus* ticks was detected by nested PCR assay targeting the *gltA* gene. In general, a total of 12 out of 247 (4.86%) *I. granulatus* ticks were detected with *Rickettsia* infection from Kinmen Island (Table 2). Based on the life stage of ticks, the *Rickettsia* infection was detected in nymphs, males and females of *I. granulatus* ticks with an infection rate of 3.81%, 0% and 6.84%, respectively (Table 2). The geographical prevalence of *Rickettsia* infection was detected only in Kinhu (7.21%) and Kinsha (3.92%) townships (Table 2).

### 3.2. Sequence Alignment and Genetic Analysis of Rickettsia Detected in Ticks

To clarify the genetic identity of *Rickettsia* in *I. granulatus* ticks of Taiwan, the sequences of *gltA* gene fragments from four Taiwan strains of *Rickettsia* performed by this study were aligned and compared with the downloaded sequences of 19 other *Rickettsia* strains from different biological and geographical origin documented in GenBank. Results indicate that all these *Rickettsia* strains detected in *I. granulatus* ticks of Taiwan were genetically affiliated to the genospecies of *R. felis* and *R. parkeri* with the highly sequence similarity of 96.8–100% and 98.7–99.2% respectively (Table 3).

In addition, intra- and inter-species analysis based on the genetic distance (GD) values of *gltA* gene indicated a lower levels (GD < 0.032 and <0.013) of genetic divergence within the *Rickettsia* strains of Taiwan, as compared with the type strain of *R. felis* and *R. parkeri*, respectively (Table 3). Further analysis based on the GD values of *ompB* gene indicated a 100% similarity of genetic divergence (GD < 0.000) within the *Rickettsia* strains of Taiwan, as compared with the IG-1 strain (GD < 0.016) from Lanyu of Taiwan and the type strain of *R. parkeri* (GD < 0.024), respectively (Table 4).

### 3.3. Phylogenetic Analysis of Rickettsia Detected in Ticks

Phylogenetic relationships based on the sequence alignment of *gltA* and *ompB* genes were performed to demonstrate the genetic relationships among 23 and 16 strains of *Rickettsia* investigated in this study, respectively. Phylogenetic trees constructed by neighbour-joining (NJ) and maximum likelihood (ML) methods were used to analyze the phylogenetic relationships of *Rickettsia* strains. Bootstrap analysis was used to analyze the repeatability of the clustering of specimens represented in phylogenetic trees.

## 4. Discussion

**Figure 2:**
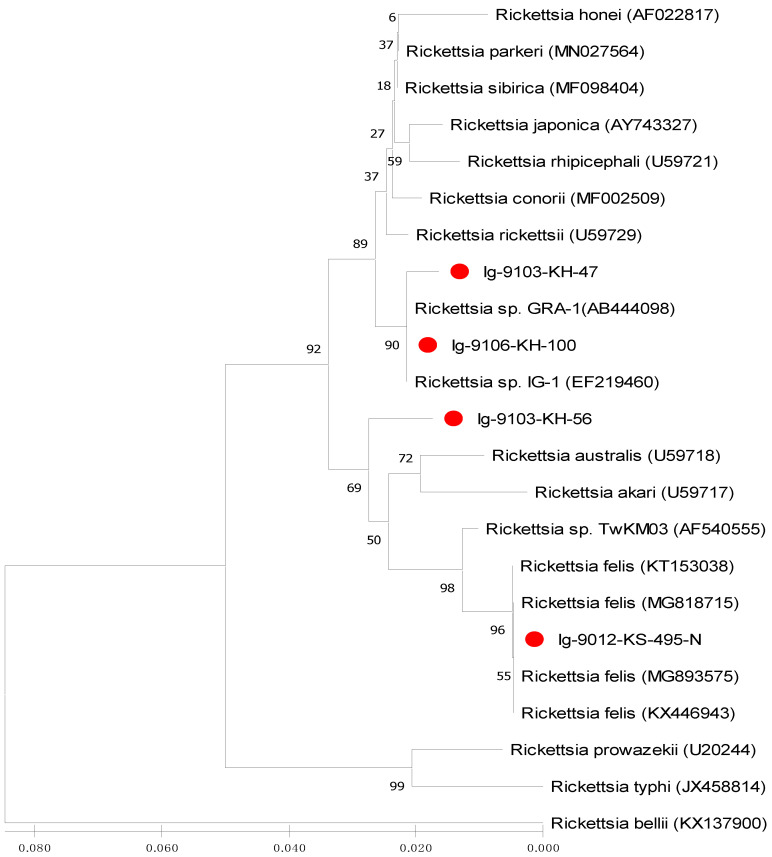
Phylogenetic relationships based on the citrate synthase gene (*gltA*) sequences of *Rickettsia* between 4 specimens (indicated as ●) collected from *Ixodes granulatus* ticks of Kinmen Island and 19 other *Rickettsia* specimens identified from various biological and geographical origins. The tree was constructed and analyzed by neighbour-joining (NJ) method using 1000 bootstraps replicates. Numbers at the nodes indicate the percentages of reliability of each branch of the tree. Branch length is drawn proportional to the estimated sequence divergence.

**Figure 3:**
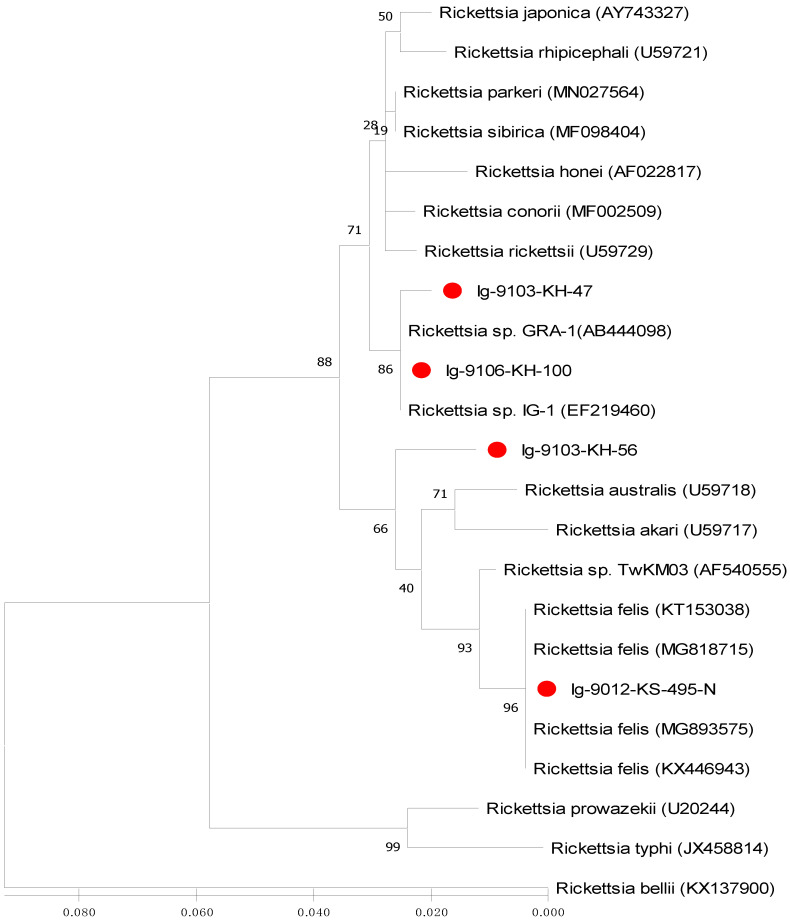
Phylogenetic relationships based on the citrate synthase gene (*gltA*) sequences of *Rickettsia* between 4 specimens (indicated as ●) collected from *Ixodes granulatus* ticks of Kinmen Island and 19 other *Rickettsia* specimens identified from various biological and geographical origins. The tree was constructed and analyzed by maximum likelihood (ML) method using 1000 bootstraps replicates. Numbers at the nodes indicate the percentages of reliability of each branch of the tree. Branch length is drawn proportional to the estimated sequence divergence.

**Figure 4:**
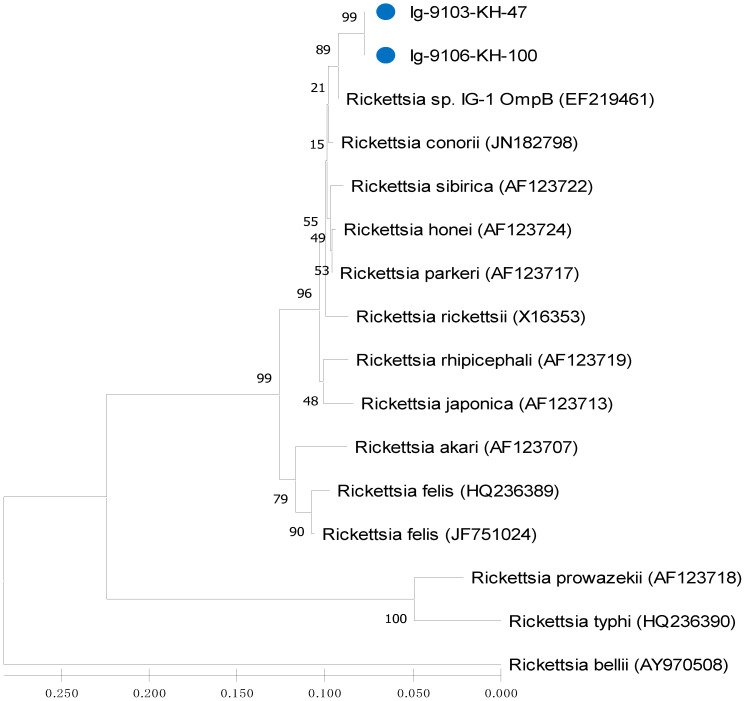
Phylogenetic relationships based on the outer membrane protein B (*ompB*) sequences of *Rickettsia* between 2 specimens (indicated as ●) collected from *Ixodes granulatus* ticks of Taiwan and 14 other *Rickettsia* specimens identified from various biological and geographical origins. The tree was constructed and analyzed by neighbour-joining (NJ) method using 1000 bootstraps replicates. Numbers at the nodes indicate the percentages of reliability of each branch of the tree. Branch length is drawn proportional to the estimated sequence divergence.

This study reports the first molecular detection and genetic identification of *Rickettsia* infection in *I. granulatus* ticks of Taiwan. In this study, the *Rickettsia* species detected in *I. granulatus* ticks from Taiwan are genetically affiliated to the genospecies of *R. parkeri* and *R. felis*, which is different from the previous studies which described a *R. honei*-like organism in *I. granulatus* ticks identified from Thailand, Taiwan and Japan [20,30,31]. Although the *R. felis* was first reported in *I. granulatus* ticks from Taiwan (Figure 2, Figure 3 and Figure 4), it had been detected in the scrub typhus mite (*Leptotrombidium deliense*) in Taiwan [38]. In addition, the *R. parkeri* is considered as an emerging tick-borne pathogen of Spotted Fever Group *Rickettsia* and it is mainly transmitted by *Amblyomma* ticks in South America (Uruguay, Argentina and Brazil) [39]. Thus, our study demonstrates the first molecular evidence confirming the presence of *Rickettsia* species detected in *I. granulatus* ticks of Taiwan and provides the first convincing sequences (GenBank accession numbers: MT847612-4 and MT847619) of *Rickettsia* species discovered in *I. granulatus* ticks of Taiwan.

The confirmed mechanism for the transmission of *R. felis* (flea/mite-borne) into ticks remains elusive. Due to the ability of parasitizing on the same rodent hosts, it is possible that the horizontal transmission of *Rickettsia* occurred between tick-flea or tick-mite interactions. Actually, ticks may accidentally feed on rodents that were previously fed by infected fleas/mites, and these ticks may acquire the *Rickettsia* infection through feeding blood from the same parasitized hosts [40]. Another non-systemic mode of transmission by a co-feeding mechanism may also contribute to the ticks feeding closely with another infected flea/mite on the same host that may facilitate the transmission of pathogens from an infected vector to a new vector [41]. Due to close contact with humans, these observations may highlight the epidemiological significance of rodents serving as a sentinel and reservoir host for the *Rickettsia* transmission in nature.

Phylogenetic relationships among *Rickettsia* in *I. granulatus* ticks can be determined by analyzing the sequence homogeneity of the *gltA* gene of *Rickettsia*. Indeed, sequence analysis based on the *gltA* gene of *Rickettsia* strains among various species from different origins had been shown to be useful for evaluating the genetic relatedness of *Rickettsia* detected from various biological and geographical sources [16,22,23,32,33]. Although the *Rickettsia* has been divided into various genospecies, most *Rickettsia* species are primarily found in arthropod hosts [33]. In this study, the phylogenetic analysis based on the sequences of *gltA* gene from *I. granulatus* ticks of Taiwan demonstrated a highly genetic homogeneity affiliated to the genospecies of *R. parkeri* and *R. felis* (Figure 2 and Figure 4, Table 3). The *R. parkeri* strains are mainly associated with the *Rickettsia* strain from humans (GenBank accession no. MN027564) and the *R. felis* strains are mainly affiliated to the *Rickettsia* strains from cat flea and lice (GenBank accession no. MG893575 and MG818715)). The phylogenetic trees constructed by either NJ or ML analysis strongly support the discrimination recognizing the separation of different genospecies between the *Rickettsia* strains in *I. granulatus* ticks collected from Taiwan and other genospecies of *Rickettsia* from different geographic and biological origins. Accordingly, these results demonstrate that genetic identities of *Rickettsia* strains detected in *I. granulatus* ticks collected from Taiwan were verified as a monophyletic group affiliated to the spotted fever (*R. parkeri*) and transitional (*R. felis*) groups of *Rickettsia*.

Whether global climate change may also increase the expansion of *Ixodes* ticks, which may enhance the transmission of tick-borne pathogens in Taiwan, remains unknown. Indeed, a previous 10-year study on rickettsias conducted in Germany demonstrated that the *Rickettsia* infection rate significantly increased over the years from 33.3% in 2005 to 50.8% in 2015 [10]. In addition, the previous study also discovered that the *I. ricinus* tick is reported to have spread into the previously unidentified northern areas of Sweden, Finland and Norway [42,43]. Because of the close association of *I. granulatus* ticks with residential area of humans, there is a serious concern regarding whether the *Rickettsia* species within this tick species can be transmitted into humans.

In this study, the *Rickettsia* infection in *I. granulatus* ticks was detected only in the Kinhu (KH) and Kinsa (KS) townships. The collection sites of KH and KS are mainly vegetable gardens as compared to the grassy areas in Kining and Kincheng townships. This difference of habitat may contribute to the capture rate of rodents in relation to the collection number of *I. granulatus* ticks from rodent hosts (Table 2). In addition, there is no detection of *Rickettsia* infection in male ticks is attributed to the small sample size of collected males. Indeed, the male tick is rarely collected from captured rodents and this phenomenon requires further investigation.

Although our results have demonstrated the PCR detection of *Rickettsia* infection in *I. granulatus* ticks, the vector competence should be further confirmed by cultivating of *Rickettsia* organisms from this tick species. Nevertheless, further investigations on the identification of vector ticks and genetic diversity of tick-borne *Rickettsia* may help to illustrate the spread of vector ticks and the risk of transmission of tick-borne rickettsial infections in Taiwan.

## 5. Conclusions

This study provides the first genetic identification of the *Rickettsia* infection detected in *I. granulatus* ticks collected from Kinmen Island of Taiwan. Based on the phylogenetic analyses, the *I. granulatus* ticks were found infected with the genospecies of *R. parkeri*-like and *R. felis*. Further investigations on the vector competence of these ticks may help to understand the potential risk and threat to human populations in Taiwan.

## Figures and Tables

**Table 1:** Phylogenetic analysis based on *gltA* and *ompB* genes of *Rickettsia* used in this study.

Genospecies/Strain	Origin of *Rickettsia* Strain		Gene Accession Number ^a^	
	Biological	Geographic	*gltA*	*ompB*
Taiwan strains				
Ig-9103-KH-56	*Ixodes granulatus*	Kinmen, Taiwan	MT847612	
Ig-9103-KH-47	*Ixodes granulatus*	Kinmen, Taiwan	MT847613	MZ198103
Ig-9106-KH-100	*Ixodes granulatus*	Kinmen, Taiwan	MT847614	MZ198104
Ig-9012-KS-495-N	*Ixodes granulatus*	Kinmen, Taiwan	MT847619	
*Rickettsia parkeri*	Human skin	Brazil	MN027564	
*Rickettsia parkeri*	Unknown	France		AF123717
*Rickettsia sibirica*	*Hyalomma asiaticum*	China	MF098404	
*Rickettsia sibirica*	Unknown	France		AF123722
*Rickettsia honei*	Unknown	Australia	AF022817	
*Rickettsia honei*	Unknown	France		AF123724
*Rickettsia conorii*	*Rhipicephalus turanicus*	China	MF002509	
*Rickettsia conorii*	Human blood	Italy		JN182798
*Rickettsia rickettsii*	*Dermacentor andersoni*	Montana, USA	U59729	
*Rickettsia rickettsii*	*Dermacentor andersoni*	Montana, USA		X16353
*Rickettsia* sp. GRA-1	*Ixodes granulatus*	Japan	AB444098	
*Rickettsia* sp. IG-1	*Ixodes granulatus*	Lanyu, Taiwan	EF219460	EF219461
*Rickettsia japonica*	Unknown	Korea	AY743327	
*Rickettsia japonica*	Unknown	France		AF123713
*Rickettsia rhipicephali*	*Rhipicephalus sanguineus*	USA	U59721	
*Rickettsia rhipicephali*	Unknown	France		AF123719
*Rickettsia akari*	Human	New York, USA	U59717	
*Rickettsia akari*	Unknown	France		AF123707
*Rickettsia australis*	Human	Australia	U59718	
*Rickettsia* sp. TwKM03	*Ixodes granulatus*	Taiwan	AF540555	
*Rickettsia felis*	Cat flea	Malta	MG893575	
*Rickettsia felis*	Lice	China	MG818715	
*Rickettsia felis*	Cat flea	Brazil	KT153038	
*Rickettsia felis*	Rat flea	Brazil	KX446943	
*Rickettsia felis*	Flea	South Korea		HQ236389
*Rickettsia felis*	*Rhipicephalus sanguineus*	Chile		JF751024
*Rickettsia prowazekii*	Beetle	France	U20244	
*Rickettsia prowazekii*	Unknown	France		AF123718
*Rickettsia typhi*	Human blood	Mexico	JX458814	
*Rickettsia typhi*	Flea	Korea		HQ236390
*Rickettsia bellii*	*Ixodes loricatus*	Brazil	KX137900	
*Rickettsia bellii*	Unknown	France		AY970508

^a^ GenBank accession numbers for *gltA* (MT847612-614 and MT847619) and *ompB* (MZ198103-4) were submitted by this study.

**Table 2:** Detection of *Rickettsia* infection in various life-cycle stages of *Ixodes granulatus* ticks collected from various sites of Kinmen Island, Taiwan by nested-PCR assay targeting the citrate synthase (*gltA*) gene of *Rickettsia*.

Site of Collection ^a^	*Rickettsia* Infection Detected in Life-Stage of Tick			Total
	Nymph	Male	Female	No. Infected /No. Examined (%)
	No. Infected /No. Examined (%)	No. Infected /No. Examined (%)	No. Infected /No. Examined (%)	
KH	1/46 (2.17)	0/14 (0)	7/51 (13.73)	8/111 (7.21)
KS	3/36 (8.33)	0/9 (0)	1/57 (1.75)	4/102 (3.92)
KN	0/5 (0)	0/0 (0)	0/3 (0)	0/8 (0)
KC	0/18 (0)	0/2 (0)	0/6 (0)	0/26 (0)
Total	4/105 (3.81)	0/25 (0)	8/117 (6.84)	12/247 (4.86)

^a^ Townships of KH (Kinhu), KS (Kinsha), KN (Kinning) and KC (Kincheng).

**Table 3:** Intra- and inter-species analysis of genetic distance values ^a^ based on the *gltA* gene sequences between *Rickettsia* strains of Taiwan and other strains of *Rickettsia* documented in GenBank.

*Rickettsia* Strains	1	2	3	4	5	6	7	8	9	10	11	12	13	14	15	16	17
1. Ig-9106-KH-100 (Taiwan)	—																
2. Ig-9103-KH-47 (Taiwan)	0.005	—															
3. *Rickettsia* sp. IG-1 (EF219460)	0.000	0.005	—														
4. *Rickettsia* sp. GRA-1 (AB444098)	0.000	0.005	0.000	—													
5. *Rickettsia parkeri* (MN027564)	0.008	0.013	0.008	0.008	—												
6. *Rickettsia sibirica* (MF098404)	0.008	0.013	0.008	0.008	0.000	—											
7. *Rickettsia conorii* (MF002509)	0.013	0.019	0.013	0.013	0.005	0.005	—										
8. *Rickettsia japonica* (AY743327)	0.016	0.021	0.016	0.016	0.008	0.008	0.013	—									
9. *Rickettsia honei* (AF022817)	0.020	0.025	0.020	0.020	0.014	0.014	0.020	0.023	—								
10. *Ig-9103-KH-56* (Taiwan)	0.027	0.032	0.027	0.027	0.029	0.029	0.035	0.032	0.043	—							
11. *Rickettsia australis* (U59718)	0.041	0.046	0.041	0.041	0.032	0.032	0.038	0.035	0.049	0.035	—						
12. *Rickettsia* TwKM03 (AF540555)	0.038	0.043	0.038	0.038	0.035	0.035	0.041	0.038	0.049	0.027	0.030	—					
13. Ig-9012-KS-495-N (Taiwan)	0.043	0.049	0.043	0.043	0.040	0.040	0.046	0.043	0.055	0.032	0.035	0.011	—				
14. *Rickettsia felis* (MG893575)	0.043	0.049	0.043	0.043	0.040	0.040	0.046	0.043	0.055	0.032	0.035	0.012	0.000	—			
15. *Rickettsia felis* (MG818715)	0.043	0.049	0.043	0.043	0.040	0.040	0.046	0.043	0.055	0.032	0.035	0.011	0.000	0.000	—		
16. *Rickettsia typhi* (JX458814)	0.084	0.090	0.084	0.084	0.081	0.081	0.081	0.078	0.099	0.081	0.093	0.081	0.087	0.087	0.087		—
17. *Rickettsia bellii* (KX137900)	0.147	0.147	0.147	0.147	0.147	0.147	0.149	0.150	0.162	0.161	0.154	0.161	0.167	0.167	0.167	0.164	0.171

^a^ The pairwise distance calculation was performed by the method of Kimura 2-parameter, as implemented in MEGA X (Kumar et al., 2018).

**Table 4:** Intra- and inter-species analysis of genetic distance values ^a^ based on the *ompB* gene sequences between *Rickettsia* strains of Taiwan and other strains of *Rickettsia* documented in GenBank.

*Rickettsia* Strains	1	2	3	4	5	6	7	8	9	10	11	12	13	14	15	16
1. Ig-9106-KH-47 (Taiwan)	—															
2. Ig-9103-KH-100 (Taiwan)	0.000	—														
3. *Rickettsia* sp. IG-1 (EF219461)	0.016	0.016	—													
4. *Rickettsia parkeri* (AF123717)	0.024	0.024	0.008	—												
5. *Rickettsia conorii* (JN182798)	0.024	0.024	0.008	0.005	—											
6. *Rickettsia honei* (AF123724)	0.026	0.026	0.010	0.003	0.008	—										
7. *Rickettsia sibirica* (AF123722)	0.032	0.032	0.016	0.008	0.013	0.010	—									
8. *Rickettsia rickettsia* (X16353)	0.034	0.034	0.018	0.016	0.016	0.018	0.024	—								
9. *Rickettsia rhipicephali* (AF123719)	0.043	0.043	0.029	0.026	0.021	0.029	0.029	0.037	—							
10. *Rickettsia japonica* (AF123713)	0.046	0.046	0.030	0.027	0.027	0.030	0.030	0.038	0.032	—						
11. *Rickettsia felis* (JF751024)	0.068	0.068	0.051	0.048	0.049	0.051	0.057	0.059	0.062	0.066	—					
12. *Rickettsia felis* (HQ236389)	0.070	0.070	0.064	0.061	0.059	0.064	0.070	0.073	0.067	0.080	0.011	—				
13. *Rickettsia akari* (AF123707)	0.087	0.087	0.070	0.067	0.069	0.070	0.076	0.078	0.079	0.086	0.040	0.047	—			
14. *Rickettsia prowazekii* (AF123718)	0.356	0.356	0.341	0.333	0.343	0.333	0.332	0.342	0.333	0.351	0.313	0.321	0.321	—		
15. *Rickettsia typhi* (HQ236390)	0.375	0.375	0.366	0.357	0.365	0.357	0.356	0.349	0.357	0.377	0.332	0.330	0.362	0.077	—	
16. *Rickettsia bellii* (AY970508)	0.484	0.484	0.478	0.468	0.468	0.474	0.472	0.470	0.475	0.468	0.471	0.477	0.487	0.546	0.563	—

^a^ The pairwise distance calculation was performed by the method of Kimura 2-parameter, as implemented in MEGA X (Kumar et al., 2018).
